# Enzymatically Modified Low-Density Lipoprotein Is Recognized by C1q and Activates the Classical Complement Pathway

**DOI:** 10.1155/2011/376092

**Published:** 2011-03-09

**Authors:** Gérard J. Arlaud, Adrienn Biro, Wai Li Ling

**Affiliations:** ^1^Laboratoire d'Enzymologie Moléculaire, Institut de Biologie Structurale Jean-Pierre Ebel, 41 rue Jules Horowitz, 38027 Grenoble Cedex 1, France; ^2^Laboratoire de Microscopie Electronique Structurale, Institut de Biologie Structurale Jean-Pierre Ebel, 41 rue Jules Horowitz, 38027 Grenoble Cedex 1, France

## Abstract

Several studies suggest that the complement system is involved in atherogenesis. To further investigate this question, we have studied the ability of native and modified forms of LDL to bind and activate C1, the complex protease that triggers the classical pathway of complement. Unlike native LDL, oxidized (oxLDL) and enzymatically modified (E-LDL) derivatives were both recognized by the C1q subunit of C1, but only E-LDL particles, obtained by sequential treatment with a protease and then with cholesterol esterase, had the ability to trigger C1 activation. Further investigations revealed that C1q recognizes a lipid component of E-LDL. Several approaches, including reconstitution of model lipid vesicles, cosedimentation, and electron microscopy analyses, provided evidence that C1 binding to E-LDL particles is mediated by the C1q globular domain, which senses unesterified fatty acids generated by cholesterol esterase. The potential implications of these findings in atherogenesis are discussed.

## 1. Introduction

It is generally accepted that accumulation of low-density lipoproteins (LDLs) in the extracellular matrix of the blood vessels is the starting point of atherogenesis [1]. Nevertheless, how this leads to chronic inflammation and injury of the arterial wall is still poorly understood. The arterial intima is known to contain a number of oxidative agents and proteolytic enzymes, and these are thought to convert LDL particles into the lipid droplets and vesicles found during the early steps of atherogenesis [2–4]. Cell-induced oxidative modification or incubation *in vitro* with high concentrations of transition metals such as copper yields LDL derivatives endowed with atherogenic properties [5, 6]. On the other hand, lipid particles enriched in unesterified cholesterol have been isolated from the arterial intima and found to be structurally similar to the LDL derivative (E-LDL) generated *in vitro* by sequential treatment with trypsin and cholesterol esterase (CEase) [7, 8]. It has been hypothesized that the oxidative and enzymatic modifications of LDL possibly complement each other, E-LDL being involved in the early steps of atherogenesis and oxidation playing a role in the progression of the disease [9].

Complement, known to be a major arm of innate immunity against pathogens, is also emerging as a potentially important factor of atherosclerosis [10]. That LDL derivatives have the ability to activate the complement system was initially described by Seifert and coworkers, who showed that cholesterol-containing lipid particles isolated from human atherosclerotic lesions activate complement to completion [11]. It was later reported by the same group that, unlike native LDL and oxidized LDL (oxLDL), E-LDL has the ability to activate complement both directly and in a C-reactive protein- (CRP-) dependent manner [12]. Triggering of the classical pathway of complement results from binding of the C1 complex, through its recognition subunit C1q, to a variety of immune and nonimmune targets and elicits activation of its partner proteases C1r and C1s [13]. We have compared the ability of oxLDL and E-LDL derivatives to interact with the C1 complex, providing evidence that E-LDL efficiently activates C1 under conditions close to the physiological situation and is recognized by C1q [14]. More recently, we have shown that C1q recognizes E-LDL particles through unesterified fatty acids generated by cholesterol esterase treatment [15]. The aim of this paper is to briefly review these findings, which shed new light on the interactions between E-LDL and the classical pathway of complement and suggest a possible implication of these interactions in atherogenesis.

## 2. Modified Lipoproteins Differentially Bind and Activate the C1 Complex of Complement

The ability of native and modified forms of LDL to trigger activation of the C1 complex was tested *in vitro* by means of a C1 activation assay in the presence of excess C1 inhibitor in order to prevent spontaneous C1 activation [16]. Under these conditions, which are close to the physiological situation, purified native LDL did not significantly activate C1. Likewise, oxLDL had no significant activating effect at concentrations up to 1 *μ*M, even though slight activation (about 10%) was observed in the presence of CRP. In contrast, sequential treatment of native LDL with trypsin and then with CEase endowed the resulting E-LDL derivative with the ability to trigger efficient C1 activation [14]. The activation level at 1 *μ*M E-LDL reached about 60%, and activation was not sensitive to CRP. Sequential treatment of LDL with trypsin and then CEase was critical to generate a C1-activating particle, and proteases such as plasmin and proteinase *K* were as efficient as trypsin for this purpose. 

**Figure 1:**
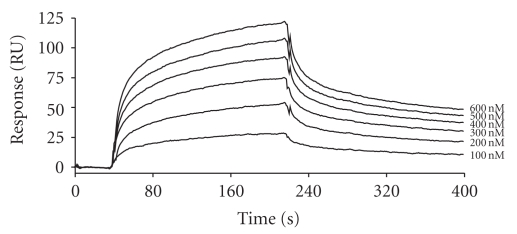
Analysis by surface plasmon resonance spectroscopy of the interaction between C1q and immobilized E-LDL. The E-LDL derivative (14,000 resonance units) was immobilized chemically on the surface of a CM5 sensor chip (GE Healthcare) and allowed to bind to increasing concentrations of soluble C1q (100–600 nM). The *K*_*D*_ value was determined from the ratio of the dissociation and association rate constants (*k*_off_/*k*_on_) (taken from [14], with permission).

The ability of C1q, the recognition unit of C1, to bind native LDL and its modified forms was investigated by surface plasmon resonance spectroscopy, using the lipoproteins as immobilized ligands and C1q as the soluble analyte. C1q did not show significant binding to native LDL but readily bound to both oxLDL and E-LDL. By recording binding curves at varying C1q concentrations, as illustrated in Figure 1 for E-LDL, the dissociation constants (*K*_*D*_) were determined. E-LDL and oxLDL each yielded values in the nanomolar range (23–75 nM), indicating that C1q binds both derivatives with high affinity [14]. 

In subsequent experiments [15], a series of proteolytic enzymes known to be present in atherosclerotic lesions were tested for their ability to generate C1-activating E-LDL particles when used in conjunction with CEase. As shown by SDS-PAGE analysis, in addition to trypsin, plasmin, kallikrein, chymase, and thrombin all extensively split the apolipoprotein B-100 (Apo-B100) of LDL, whereas tryptase, and matrix metalloprotease-2 had milder effects. Subsequent incubation with CEase revealed that samples initially treated with plasmin, thrombin, tryptase, and matrix metalloprotease-2 displayed a C1-activating ability comparable to that obtained using trypsin, whereas chymase and kallikrein had moderate or no effect. Thus, initial treatment of LDL with a protease was clearly a prerequisite to generate C1-activating particles, but there was no strict correlation between the extent of Apo-B100 degradation and the resulting C1 activation extent. Increasing the CEase concentration and the incubation time both readily increased the C1-activating ability of the resulting LDL particles, yielding complete C1 activation after incubation of trypsin-treated LDL with 320 milliunits/mL CEase for 18 h at 37°C [15]. Thus, subsequent modification of trypsin-treated LDL with CEase was clearly a determinant step for generating particles recognized by C1q and exhibiting C1-activating ability. In contrast, treatment of native LDL with phospholipase A_2_ or sphingomyelinase did not yield significant C1 activation.

## 3. C1q Recognizes a Lipid Component of E-LDL

**Figure 2:**
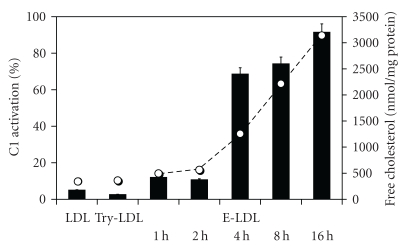
C1 activation by E-LDL particle: correlation with the amount of unesterified cholesterol generated. LDL (1 mg/mL) was treated with 20 *μ*g/mL trypsin for 2 h at 37°C and then with 320 milliunits/mL CEase for the indicated periods at 37°C. The lipid fraction from each sample was extracted and incorporated into vesicles. Each vesicle was tested for its C1-activating ability (black bars). The cholesterol content of each lipid fraction (open circles) was determined by reverse-phase HPLC. LDL: native LDL; Try-LDL: trypsin-treated LDL (taken from [15], with permission).

As a first step towards identification of the LDL component(s) recognized by C1q, the lipid fractions of native LDL, trypsin-treated LDL, and E-LDL were extracted and used to prepare vesicles which were then tested for their ability to activate C1 [15]. As shown in Figure 2, vesicles containing the lipid fraction of unmodified or trypsin-treated LDL did not yield C1 activation. In contrast, vesicles prepared from LDL samples treated with trypsin and then incubated with CEase for increasing periods at 37°C developed increasing C1-activating ability, to reach about 90% activation after incubation with 320 milliunits CEase for 16 h. In contrast, the protein component of this sample left after lipid extraction did not induce C1 activation. Likewise, native or trypsin-treated ApoB-100 had no activating effect.

As measured by reverse-phase HPLC, the lipid fraction of native LDL contained a small amount of unesterified cholesterol (398 ± 50 nmol/mg of protein), as well as the cholesteryl esters characteristic of LDL (22 : 6, 20 : 4, 18 : 2, 18 : 1, and 16 : 0). Incubation overnight at 37°C led to nearly complete disappearance of the cholesteryl ester peaks, with concomitantly a raise of unesterified cholesterol to 3140 ± 160 nmol/mg of protein. Analysis of the lipid fraction of the E-LDL particles generated upon incubation for increasing periods with CEase showed a good correlation between the content in unesterified cholesterol and the extent of C1 activation. This suggested that cholesterol itself, or fatty acids, or both of these molecules resulting from CEase treatment could be recognized by C1q.

## 4. C1q Recognizes E-LDL through Unesterified Fatty Acids Generated by CEase

To identify the lipid component(s) of E-LDL recognized by C1q, E-LDL was treated with methyl-*β*-cyclodextrin (MBCD), a reagent known to extract unesterified cholesterol from membranes [15]. Incubation of E-LDL particles with increasing concentrations of MBCD for 1 h at 37°C progressively abolished their C1-activating ability. However, parallel analyses by thin-layer chromatography clearly indicated that MBCD not only depleted free cholesterol but also removed unesterified fatty acids, yielding in both cases >70% depletion at an MBCD concentration of 2.25 mM.

Incubation of the E-LDL particles with increasing concentrations of human serum albumin, a reagent known to remove unesterified fatty acids, decreased their C1-activating ability in a dose-dependent fashion, resulting in >60% inhibition upon treatment with 10% (w/v) albumin. Analysis by thin-layer chromatography revealed that the C1-activating ability of the samples roughly correlated with the extent of unesterified fatty acid removal, whereas free cholesterol was not depleted under these conditions. These data provided a first indication that binding of the C1q subunit of C1 to E-LDL particles involved the unesterified fatty acids generated by CEase treatment.

**Figure 3:**
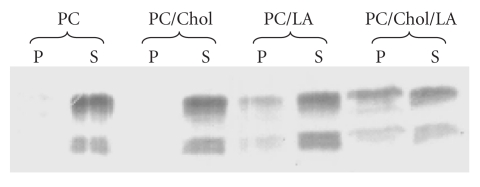
Cosedimentation analysis of the interaction between the C1q globular domain and artificial lipid vesicles. Vesicles were prepared from PC alone, PC : cholesterol (1 : 2, w/w), PC : linoleic acid (1 : 2, w/w), or PC : cholesterol : linoleic acid (1 : 2 : 2, w/w/w). Each type of vesicle was incubated with the C1q globular domain (C1q GR), and binding was measured from the relative amount of C1q GR associated with the vesicles in the ultracentrifugation pellet, as determined by SDS-PAGE analysis of the pellet (P) and supernatant (S) fractions. Chol: cholesterol; LA: linoleic acid; PC: phosphatidylcholine (taken from [15], with permission).

To further investigate this question, model vesicles containing phosphatidylcholine (PC) and increasing proportions of unesterified cholesterol and/or linoleic acid were prepared and tested for their ability to activate C1 [15]. Increasing the amount of linoleic acid increased the C1-activating potential of the vesicles, yielding about 65% activation at a 1 : 1 linoleic acid : PC ratio, and then had the opposite effect at a 2 : 1 ratio. In contrast, increasing both linoleic acid and cholesterol dose-dependently increased the ability of the vesicles to activate C1, yielding full activation at a 2 : 2 : 1 linoleic acid : cholesterol : PC ratio. Further analyses were conducted using lipid vesicles containing PC plus either cholesterol, or linoleic acid, or both cholesterol and linoleic acid. After incubation of these vesicles with the C1q globular domain (C1q GR), interaction was assessed by cosedimentation analysis from the relative amount of C1q GR sedimenting with the vesicles in the ultracentrifugation pellet (Figure 3). No binding to vesicles containing PC alone or PC + cholesterol was detected. A slight but significant binding (about 20%) was observed using vesicles containing PC + linoleic acid, and this value increased to 46% when both cholesterol and linoleic acid were present (Figure 3). Analysis of the pellets revealed that linoleic acid was more efficiently incorporated into the vesicles in the presence of cholesterol. It became clear, therefore, that linoleic acid was the only ligand recognized by C1q, the enhancing effect exerted by cholesterol being due to its ability to facilitate incorporation of free fatty acids into the vesicles. Another indication from the cosedimentation experiments was that recognition by C1q of the fatty acids of E-LDL was mediated by its globular (GR) domains.

## 5. Electron Microscopy of E-LDL-Bound C1q Molecules

**Figure 4:**
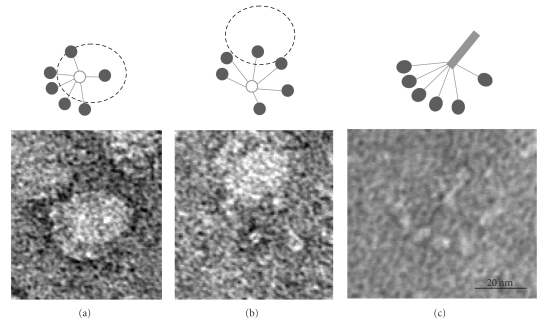
Electron micrographs of E-LDL-bound C1q molecules. (a, b) Examples of C1q molecules interacting through most of their globular domains (a) or only a few (b). (c) Representative example of a free C1q molecule (modified from [15], with permission).

Negative staining electron microscopy was used to visualize interaction of the whole C1q molecule with E-LDL particles. Bound C1q molecules were clearly seen to interact with the particles through their globular domains. In some cases, most of the 6 globular domains were found to follow the curvature of the particles (Figure 4(a)), whereas other C1q molecules interacted through only a few heads (Figure 4(b)). Some free C1q molecules not interacting with E-LDL particles could also be identified (Figure 4(c)).

## 6. Discussion

The various experiments carried out in the past years provide clear experimental evidence that, in contrast to native LDL and oxLDL, E-LDL is endowed with the ability to activate C1, the multimolecular protease that triggers the classical pathway of complement. The observation that native LDL particles do not activate C1 is consistent with the fact that these are not recognized by C1q. In contrast, oxLDL particles are sensed by C1q, with a binding affinity similar to that determined for E-LDL; however, they do not trigger C1 activation. It appears likely therefore that oxidation generates C1q binding sites on the LDL surface, but these are distributed in a manner that does not allow C1 activation. An alternative explanation is that C1 activation by oxLDL is efficiently prevented by C1 inhibitor, as observed for other “weak” nonimmune activators of C1 such as DNA and heparin [17, 18]. In contrast, the fact that E-LDL behaves as a potent C1 activator in the presence of excess C1 inhibitor strongly suggests that this process observed *in vitro* is biologically relevant.

A further major conclusion from these investigations is that C1 binding to E-LDL particles is mediated by the C1q globular domains and that these recognize the unesterified fatty acid molecules generated by CEase treatment. The conversion of native LDL into a particle endowed with C1-activating ability clearly requires prior treatment with a proteolytic enzyme and then with CEase. In addition to trypsin used as a model, several proteases (plasmin, matrix metalloproteases-2, thrombin, tryptase) present in the atherosclerotic lesions [19, 20] are able to generate E-LDL particles with high C1-activating ability. Considering that CEase itself is present in the arterial intima [8, 21], it appears likely that C1-activating E-LDL particles are indeed generated *in vivo*. In agreement with this hypothesis, electron microscopy analyses show that extensive hydrolysis of cholesteryl esters by CEase generates large liposome-like particles [15] similar to those previously described by Chao et al. [8] and reminiscent of the lipid particles observed in atherosclerotic lesions [7, 22].

Proteolysis of ApoB-100 appears necessary to allow access of cholesteryl ester molecules to CEase. Although no strict correlation was observed between the extent of ApoB-100 degradation and the C1-activating ability of the resulting particle, it should be emphasized that the SDS-PAGE technique used only gives a rough account of the extent of LDL degradation. The most likely hypothesis is therefore that proteolytic treatment of the LDL particle disorganizes ApoB-100 [8], hence allowing CEase to gain access to the underlying hydrophobic core containing cholesteryl esters and triglycerides, resulting in the production of large amounts of cholesterol and unesterified fatty acids.

How C1q senses fatty acids on the E-LDL surface remains to be determined at the molecular level. However, C1q binding is clearly mediated by its globular domain, indicating that, in addition to its numerous ligands already identified [23], this domain recognizes the polar head of fatty acids and therefore possesses a binding site for carboxyl groups. This is consistent with the known ability of C1q to bind polyanionic ligands [24], considering that treatment with CEase converts LDL particles into polyanions. These investigations provide clear evidence that E-LDL is recognized by C1q, further establishing the role of this protein as a sensor of altered self-components. Another modified form of LDL, acetylated LDL, was recently shown to be recognized by C1q, as well as by two other innate immune recognition proteins involved in activation of the lectin, pathway of complement, mannan-binding lectin, and L-ficolin [25, 26].

The observation that E-LDL triggers activation of the classical complement pathway provides further support for an implication of complement in atherogenesis. It now remains to be established whether complement activation has a protective effect through the removal of E-LDL particles from atherosclerotic plaques or is proatherogenic because of the intrinsic ability of the terminal complement pathway to generate inflammatory mediators.
